# Epilepsy phenotype and gene ontology analysis of the 129 genes in a large neurodevelopmental disorders cohort

**DOI:** 10.3389/fneur.2023.1218706

**Published:** 2023-08-14

**Authors:** Young Jun Ko, Soo Yeon Kim, Seungbok Lee, Jihoon G. Yoon, Man Jin Kim, Hyeji Jun, Hunmin Kim, Jong-Hee Chae, Ki Joong Kim, Kwangsoo Kim, Byung Chan Lim

**Affiliations:** ^1^Department of Pediatrics, Chung-Ang University Gwangmyeong Hospital, Gwangmyeong, Republic of Korea; ^2^Department of Pediatrics, Pediatric Neuroscience Center, Seoul National University Children's Hospital, Seoul, Republic of Korea; ^3^Department of Genomic Medicine, Seoul National University Hospital, Seoul, Republic of Korea; ^4^Biomedical Research Institute, Seoul National University Hospital, Seoul, Republic of Korea; ^5^Department of Pediatrics, Seoul National University Bundang Hospital, Seongnam, Republic of Korea

**Keywords:** neurodevelopmental disorders, epilepsy, genetic testing, gene ontology, seizure

## Abstract

**Objective:**

Although pediatric epilepsy is an independent disease entity, it is often observed in pediatric neurodevelopmental disorders (NDDs) as a major or minor clinical feature, which might provide diagnostic clues. This study aimed to identify the clinical and genetic characteristics of patients with epilepsy in an NDD cohort and demonstrate the importance of genetic testing.

**Methods:**

We retrospectively analyzed the detailed clinical differences of pediatric NDD patients with epilepsy according to their genetic etiology. Among 1,213 patients with NDDs, 477 were genetically diagnosed by exome sequencing, and 168 had epilepsy and causative variants in 129 genes. Causative genes were classified into two groups: (i) the “epilepsy-genes” group resulting in epilepsy as the main phenotype listed in OMIM, Epi25, and ClinGen (67 patients) and (ii) the “NDD-genes” group not included in the “epilepsy-genes” group (101 patients).

**Results:**

Patients in the “epilepsy-genes” group started having seizures, often characterized by epilepsy syndrome, at a younger age. However, overall clinical features, including treatment responses and all neurologic manifestations, showed no significant differences between the two groups. Gene ontology analysis revealed the close interactions of epilepsy genes associated with ion channels and neurotransmitters.

**Conclusion:**

We demonstrated a similar clinical presentation of different gene groups regarding biological/molecular processes in a large NDDs cohort with epilepsy. Phenotype-driven genetic analysis should cover a broad scope, and further studies are required to elucidate integrated pathomechanisms.

## 1. Introduction

Epilepsy is a common neurologic disorder with a high incidence in childhood (1). Children with epilepsy have different comorbidities, such as especially neurodevelopmental disorders (NDDs), including intellectual disability (ID), autism spectrum disorder (ASD), and movement and behavioral symptoms (2). Several genes and environmental factors are associated with epilepsy and NDDs, and patients show overlapping and heterogeneous symptoms and clinical courses. An epileptic seizure is the most common distinct phenotype and is often the first symptom of NDDs. Over 30% of patients with ASD are estimated to have epilepsy (3), and ~20–50% of children with epilepsy have NDDs (4, 5).

Traditionally, epilepsy was defined and classified according to seizure semiology and electrophysiologic profiles, and accompanying disabilities were considered comorbidities. However, the concept of childhood-onset epilepsy has changed with advances in genetic testing. Epileptic encephalopathy, defined after the 2000s, is a group of disorders presenting as frequent seizures, electrophysiologic abnormalities, and various cognitive dysfunctions from early childhood (6). Disease boundaries have expanded even further as NDD patients with or without epilepsy share the same genetic etiology (7). Some children with causative variants in epilepsy genes may show intractable epilepsy as the main feature, leading to developmental delay or cognitive decline; others can present early developmental problems before seizure onset. The time gap between symptom onset and confirmative diagnosis has decreased because of the easy accessibility and lower cost of next-generation sequencing (NGS). NGS shortens the diagnostic delay, allowing early diagnosis before seizure onset for more patients, which has broadened the disease spectrum and blurred the boundaries of NDDs and epilepsy (7–10). Accordingly, the concept of “developmental and epileptic encephalopathy (DEE)” has been introduced and recognized worldwide (11, 12).

NDDs are one of the most common disease entities in the pediatric neurology clinic. In NDDs, various initial symptoms often evolve into different or multiple symptoms over time. It is difficult to predict which patients will develop epilepsy in the future, and detailed prognoses are challenging to determine. NGS is an important diagnostic test for NDD patients, and its diagnostic yield varies from 5 to 90% depending on the platforms and inclusion criteria (2, 13, 14). Phenotypes are essential factors for the final diagnosis after NGS, and seizures in patients with non-specific NDDs can provide an important clue for the final diagnosis. However, the number of seizure-associated phenotypes for which the molecular basis is known has been increasing, and over 1,500 phenotypes have recently been reported (15). Similar to patients with NDDs, patients with “traditional” epilepsy genes commonly show various neurologic symptoms before seizure onset similar to patients with NDDs. These observations suggest the limitation of the clinical approach based on curated epilepsy-annotated genes.

In this study, we aimed to thoroughly examine the clinical differences of pediatric NDD patients with epilepsy according to their genetic etiology and demonstrate the relevance of clinical genetic testing.

## 2. Materials and methods

### 2.1. Patient enrollment and study approval

We initially selected patients who visited the pediatric neurology clinic of Seoul National University Children's Hospital between January 2011 and December 2021 with the following inclusion criteria: (1) clinically diagnosed with NDDs based on the Diagnostic and Statistical Manual of Mental Disorders, 5th Edition (DSM-V) criteria (16); (2) diagnosed with epilepsy according to the 2014 clinical definition of epilepsy by the International League Against Epilepsy (ILAE) during the entire follow-up period (7, 17); and (3) underwent exome sequencing (ES) for molecular diagnosis. Patients with a possible secondary etiology or diagnosed by other genetic tests were excluded.

This study was approved by the institutional review board (IRB) of Seoul National University Hospital (IRB Nos. 1101-110-353, 1406-081-588, and 1904-054-1027).

### 2.2. Review of medical records

To examine clinical features in detail and conduct further analyses, we reviewed the entire medical records, including perinatal history, detailed developmental milestones, family history, detailed information on epilepsy (onset age, syndromic diagnosis, or fever sensitivity), growth profiles, cognitive function with objective test results, social performances, other neurologic symptoms (ataxia, dyskinesia, hypotonia, stereotyped movement, or spasticity), minor anomalies, facial dysmorphisms, and diagnostic test results. Epilepsy syndrome was classified according to the guidelines of the ILAE (7).

### 2.3. Exome sequencing and variant annotation

ES was performed at the Seoul National University Hospital between 2015 and 2021, and the detailed process has been described in a previous study (18). Capture probes targeting the entire exonic regions based on SureSelect Human All Exon V5 (Agilent Technologies, Santa Clara, CA, USA) were used, except for five patients using V6. The library was prepared according to the manufacturer's instructions. Paired-end sequencing was performed with the HiSeq 2500 sequencing system (Illumina, San Diego, CA, USA). The sequence reads were aligned to the Consortium Human Build 37 (patch release 13) using the Burrows–Wheeler Aligner (v. 0.7.17). Picard software (v. 2.9.0), SAMtools (v. 1.9), and the genome analysis toolkit (v. 4.1.2) were used for the removal of duplicates, realignment, and base recalibration. Variants were called using GATK HaplotypeCaller in the GVCF mode and were annotated using SnpEff, ANNOVAR, and InterVar. The pathogenicity of variants was evaluated according to the American College of Medical Genetics (ACMG) standard guidelines (19). For the patients with only variants of unknown significance, a re-analysis of ES data was performed every 6 months to 1 year. According to the updated literature, the pathogenicity of variants has changed.

### 2.4. Classification and gene ontology analysis of causative genes

We classified annotated genes into two groups. Genes that resulted in epilepsy as the main phenotype were defined as the “epilepsy-genes” based on the following criteria: (i) genes listed in Epi25 or ClinGen (20, 21) and (ii) annotated as causative genes for epilepsy or DEE in Online Mendelian Inheritance in Man (OMIM) (15). Genes that met neither of the above criteria were classified as the “NDD-genes” group.

To elucidate the biological significance of the epilepsy genes and NDD genes, we performed gene ontology (GO) network analysis using Cytoscape (v.3.9.1) (22) software with the ClueGO plug-in (v.2.5.8) (23). ClueGO identifies enriched GO terms linked based on the kappa score and presents their interactions as a network. A two-sided (enrichment/depletion) hypergeometric test was used for the enrichment analysis. Only the GO terms with Bonferroni step-down adjusted *p*-values < 0.05 were considered significant and included in the analysis. Functionally interrelated GO terms were grouped by the same color, and the GO term with the smallest *p*-value was designated as the leading term of each group.

### 2.5. Statistical analysis

To analyze the phenotype–genotype associations in patients, we compared clinical features between the “epilepsy-genes” and “NDD-genes” groups. Numerical and ordinal data are expressed as the means or medians with the spread by standard deviations (SD) or inter-quartile ranges (IQRs), and nominal data are expressed as frequencies. Numerical and ordinal dependent variables were compared by an independent *t*-test for non-normally distributed measurements. The categorical dependent variables of the study were evaluated by multivariate logistic regression to investigate whether a specific gene group was related to particular seizure and neurologic phenotypes. An alpha value of 0.05 was considered significant. Statistical analyses were performed with IBM SPSS Statistics version 25.0 (SPSS 25.0; IBM Corp., Armonk, NY, USA).

## 3. Results

**Figure 1:**
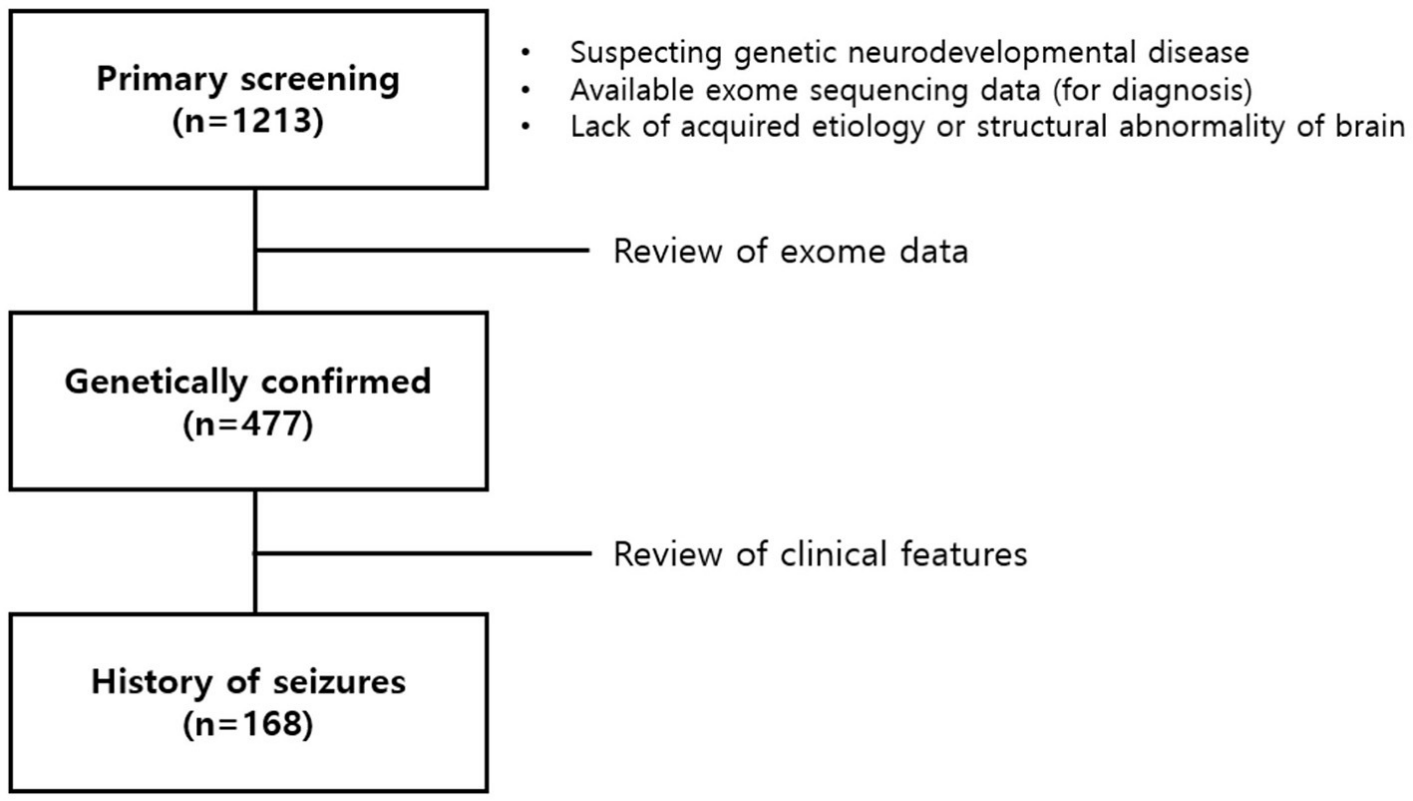
Process of patient selection in the study.

Among 1,213 patients with NDDs without acquired causes, 477 were genetically diagnosed by ES (39.3%). The diagnostic yield of ES varied across different testing approaches: singleton (215/481, 44.7%), duo (13/30, 43.3%), and trio (249/702, 35.5%). Statistical analysis using a chi-squared test revealed significant differences in the diagnostic yield among singleton, duo, and trio testing groups (*p* < 0.001). Finally, the study enrolled 168 patients with epilepsy, including 86 male patients and 82 female patients (Figure 1). Among them, 119 patients (70.8%) had undergone one or more genetic tests, which showed inconclusive results prior to ES. Chromosomal microarray (CMA), single gene tests, and target gene panel sequencing were performed for 32 (19.0%), 69 (41.1%), and 49 (29.2%) patients, respectively. ES included the proband (94/168, 56.0%), duo (2/168, 1.2%), and trio (72/168, 42.9%). Finally, 129 causative genes were identified in 168 patients. There were 44 genes identified in 67 patients in the “epilepsy-genes” group and 85 genes identified in 101 patients in the “NDD-genes” group. The complete gene list according to the classification criteria is presented in Table 1.

**Table 1:** List of genes identified in patients according to the classification.

**Group**	**Gene list**
Epilepsy genes (44 genes)	ALDH5A1, ALDH7A1, ALG13, ARX, CACNA1A, CASK, CDKL5, CLN6, COL4A1, CYFIP2, DNM1, DYNCH1, FGF12, FOXG1, GABBR2, GABRB1, GNAO1, GRIN1, GRIN2B, GRIN2D, IQSEC2, KCNB1, KCNC1, KCNCQ2, KCNT2, MECP2, PACS2, PCDH19, PIGA, PIGT, PPP3CA, SCN1A, SCN1B, SCN2A, SCN8A, SLC2A1, SLC6A1, SMC1A, SPTAN1, STXBP1, SYNGAP1, SZT2, UGDH, YWHAG
NDD genes (85 genes)	ABAT, ABCC8, ACADVL, ACO2, ACOX1, ANKRD11, ARID1B, ARSA, ASXL1, ATP6AV0A2, ATRX, BRAF, BRAT1, CAMK2A, COX15, CSNK2B, DDX3X, DEGS1, DHDDS, DLG4, DNM1L, DNMT3A, DYRK1A, EIF2AK2, EIF2B2, EIF2S3, GJA1, GLB1, GRIA2, GRIA3, HEPHL1, HDAC8, HEXA, HK1, HSD17B4, HUWE1, IARS2, ITPR1, KDM5C, KIAA1109, KIF4A, KMT2A, KMT2C, KMT2D, L1CAM, LAMA2, LONP1, MAPK8IP3, NALCN, NARS2, NDUFAF6, NDUFV1, NFIX, NSD1, OGT, OPHN1, OTUD6B, PAFAH1B1, PCYT2, PDHA1, PEX5, PIK3R2, PLA2G6, PNPT1, PPP2R5D, PRUNE1, PTPN23, RAB3GAP1, SETD2, SETD5, SLC19A3, SMC3, SNAP25, SPTBN2, ST3GAL5, TRRAP, TUBA1A, TUBB4A, TUBGCP6, UBE3A, VPS13B, VPS13D, WDFY3, WDR26, WDR81

### 3.1. Clinical features

The median age of seizure onset was 1.4 years (IQR, 0.4–5 years). The initial seizure type was generalized seizure in 96 patients (57.1%), focal seizure in 61 patients (36.3%), and undetermined in 11 patients (6.5%). In total, 44 of the 168 patients (32.1%) received a syndromic diagnosis for epilepsy based on ILAE guidelines. West syndrome (36/54, 66.7%) was the most common epilepsy syndrome, followed by Lennox–Gastaut syndrome (15/54, 27.8%), epilepsy in infancy with migrating focal seizure (4/168, 2.4%), Dravet syndrome (3/168, 1.8%), Ohtahara syndrome (1/168, 0.6%), and progressive myoclonic epilepsy (1/168, 0.6%). Half the patients (84/168, 50.0%) exhibited drug-resistant epilepsy (DRE).

Most patients (164/168, 97.6%) had neurologic symptoms in addition to seizures. Among various neurologic features, developmental delay or ID (160/168, 95.2%) was observed in almost all patients. Brain anomalies were noted in 73 patients (43.5%), including white matter abnormalities in 49 patients, gray matter abnormalities in 18 patients, malformation of cortical development in 15 patients, cerebellar atrophy in 10 patients, progressive atrophy in 7 patients, and cavernous hemangioma in 1 patient. Neonatal or infantile hypotonia was noted in 72 patients (42.9%). Furthermore, the following neurologic features were observed: microcephaly (50/168, 29.8%), regression (49/168, 29.2%), spasticity (31/168, 18.5%), ataxia (29/168, 17.3%), facial dysmorphism (24/168, 14.3%), autism (23/168, 13.7%), dyskinesia (17/168, 10.1%), and macrocephaly (16/168, 9.5%).

### 3.2. Genetic diagnosis

A total of 129 genes were identified in 168 patients. Detailed information on the variants and genes is presented in Supplementary Tables 1, 2. Approximately half of the patients (88/168, 52.4%) had causative variants in autosomal dominant (AD) genes. Autosomal recessive (AR) and X-linked (XL) genes were noted in 52 (31.0%) and 28 (16.7%) patients, respectively. Among patients with variants in epilepsy genes, AD (42/67, 62.7%) inheritance was the most prevalent, followed by XL (17/67, 25.4%) and AR (8/67, 11.9%) inheritance. Variants in NDD genes were mostly inherited in an AD (46/101, 45.5%) or AR (44/101, 43.6%) pattern, followed by an XL (11/101, 10.9%) inheritance. *De novo* variants were identified through segregation analysis in 85 patients (85/168, 50.6%).

Following the genetic diagnosis, appropriate medical interventions were implemented for certain patients. Specifically, two patients (cases 2 and 3) with *ALDH7A1* variants were recommended to continue high-dose pyridoxine supplementation. Additionally, two patients (cases 53 and 54) with intractable seizures started to follow a ketogenic diet after identifying *SLC2A1* variants. In the case of six patients with variants in other channelopathy-related genes (*SCN1A, SCN2A, SCN8A*, and *KCNQ2*), the previous literature guided the choice of anti-seizure medication. Moreover, genetic counseling was provided to patients and their families, assisting them in family planning for future pregnancies.

A total of 30 genes were repeatedly identified in 69 patients within our cohort. The detailed phenotypes of patients with variants in these 30 genes are presented in Supplementary Table 3. Specific neurodegenerative diseases, such as GM1-gangliosidosis, caused by *GLB1* or ceroid lipofuscinosis caused by *CLN6* presented a consistent clinical course of early developmental delay followed by neurologic deterioration, seizures, and generalized spasticity. However, most of these symptoms were observed at the time of ES. On the other hand, genes associated with NDDs showed a range of presentations. For instance, a patient with the *IQSEC2* variant showed intractable seizures and poor developmental outcomes, while another patient with the *IQSEC2* variant presented mild to moderate degrees of intellectual disability and well-controlled seizures.

### 3.3. Comparison of epileptic and neurologic features according to gene classification

We compared the clinical features of patients with mutations in epilepsy genes and those patients with mutations in NDD genes. Subsequently, we compared gene ontology between the two groups.

#### 3.3.1. Phenotypic differences according to gene classification

Both groups showed significant differences in the age of seizure onset, major seizure type, and syndromic classification. In particular, the median age of seizure onset showed a statistical difference between the “epilepsy-genes” and “NDD-genes” groups (12 months, IQR 4–30 months vs. 24 months, IQR 7–60 months, *p* = 0.007). However, after controlling other variables constantly, the age of seizure onset did not show a significant association with each gene group (*p* = 0.980). Generalized seizures were more prevalent in the “epilepsy-genes” group (*p* = 0.004), while focal seizures were more common in the “NDD-genes” group than in the “epilepsy-genes” group (*p* = 0.001). Epilepsy syndromes were more frequently observed in the “epilepsy-genes” group (55.2%) compared to the “NDD-genes” group (20.8%) (*p* = 0.001). However, there was no significant difference in the prevalence of drug-resistant epilepsy (DRE) between the “epilepsy-genes” and “NDD-genes” groups (56.7 vs. 44.6%, *p* = 0.173). The two groups had no significant difference in the prevalence of any neurologic features. Detailed information and statistical data regarding the seizure and neurologic features of the two groups are presented in Table 2.

**Table 2:** Epilepsy and neurologic features in epilepsy genes and NDD genes groups.

**Epilepsy features**	**Total (168 patients)**	**Epilepsy-genes (67 patients)**	**NDD genes (101 patients)**	**Coefficient (*B*)**	**Wald *X*^2^**	**Odds ratio**	***P*-value**
**Median age at seizure onset, months (IQR)**	16.8 (IQR 4.8–60)	12 (IQR 4–30)	24 (IQR 7–60)		5.988		0.980
**Seizure type at onset [*****n*** **(%)]**							
Generalized	96 (57.7%)	45 (67.2%)	51 (50.5%)		11.171		0.004
Focal	61 (36.3%)	16 (23.9%)	45 (44.6%)	1.824	10.937	6.199	0.001
Unknown	11 (6.5%)	6 (9.0%)	5 (5.0%)	0.142	0.013	1.153	0.908
**Drug resistance [*****n*** **(%)]**	84 (50.0%)	38 (56.7%)	45 (44.6%)	−0.698	1.856	0.497	0.173
**Epilepsy syndrome [*****n*** **(%)]**	58 (34.5%)	37 (55.2%)	21 (20.8%)	−1.963	14.424	0.140	0.001
Ohtahara syndrome	1 (1.5%)	1 (1.5%)	0	−0.416	0.085	0.660	0.770
EIMF	4 (1.5%)	1 (1.5%)	3 (3.0%)	0.703	0.364	2.020	0.546
Dravet syndrome	3 (4.5%)	3 (4.5%)	0	−21.659	0.000	0.000	0.999
West syndrome	35 (31.3%)	21 (31.3%)	14 (13.9%)	−1.043	7.140	0.352	0.008
LGS	14 (14.9%)	10 (14.9%)	4 (4.0%)	−1.325	4.518	0.266	0.034
PME	1 (1.5%)	1 (1.5%)	0	−0.416	0.085	0.660	0.770
**DD or ID**	160 (95.2%)	64 (95.5%)	96 (95.0%)	−0.786	0.322	0.570	0.456
**Brain anomaly**	73 (43.5%)	21 (31.3%)	52 (51.5%)	0.815	2.334	2.260	0.127
**Hypotonia**	72 (42.9%)	24 (35.8%)	48 (47.5%)	0.639	1.704	1.895	0.192
**Regression**	49 (29.2%)	18 (26.9%)	31 (30.7%)	0.337	0.344	1.401	0.558
**Microcephaly**	45/152 (29.6%)	15/60 (25.0%)	30/92 (32.6%)	0.173	0.104	1.189	0.747
**Spasticity**	31 (18.5%)	7 (10.4%)	24 (23.8%)	1.042	2.615	2.835	0.106
**Ataxia**	29 (17.3%)	12 (17.9%)	17 (16.8%)	−0.896	1.473	0.408	0.225
**Autism**	23 (13.7%)	13 (19.4%)	10 (9.9%)	−0.285	0.125	0.752	0.724
**Facial dysmorphism**	24 (14.3%)	7 (10.4%)	17 (16.8%)	0.343	0.248	1.410	0.618
**Dyskinesia**	17 (10.1%)	9 (13.4%)	8 (7.9%)	−0.829	1.070	0.436	0.301
**Macrocephaly**	17/152 (11.2%)	3/60 (5.0%)	14/92 (15.2%)	1.021	1.090	2.775	0.296

DD, developmental delay; EIMF, epilepsy of infancy with migrating focal seizures; ID, intellectual disability; LGS, Lennox–Gastaut syndrome; PME, progressive myoclonus epilepsy.

#### 3.3.2. Gene ontology analysis

**Figure 2:**
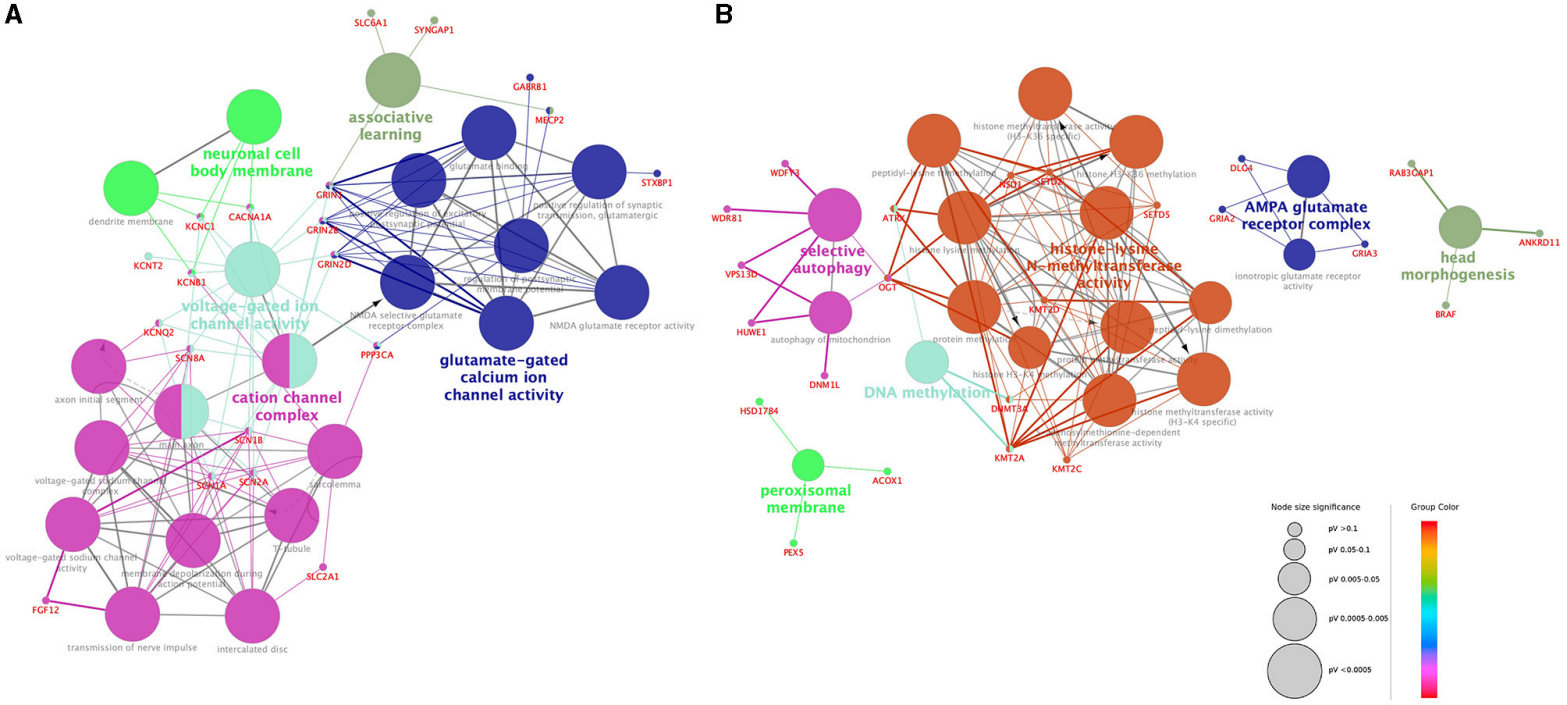
Visualization of the gene ontology and pathway network of each gene group. Functionally grouped networks of epilepsy genes **(A)** and NDD genes **(B)** were derived from ClueGO enrichment analysis. Gene ontology terms and their associated genes share the same node color. The node size of each term corresponds to its enrichment significance. The lower the adjusted *p*-value of each term, the larger the node size. Edges are created based on the kappa score (≥0.4), which is calculated by taking into account the number of genes shared between two terms. Edge thickness is proportional to the kappa score.

According to the GO network analysis of physiological pathways, epilepsy genes exhibited different patterns than NDD genes. A total of 20 epilepsy genes and 24 NDD genes showed a significant association (false discovery rate and *p*-value < 0.05). Epilepsy genes formed a complex network with each other and showed a relatively organized pattern. They demonstrated functional relationships with ion channels, neuronal cells, and neurotransmitters such as cation channel complexes, voltage-gated ion channel activity, neuronal cell body membranes, glutamate-gated calcium ion channel activity, and associative learning (Figure 2A). In contrast, NDD genes demonstrated no solid or consistent association with each other. Some fragmented associations, such as histone-lysine N-methyltransferase activity, DNA methylation, AMPA glutamate receptor complex, peroxisomal membrane, selective autophagy, and head morphogenesis, were noted among NDD genes (Figure 2B). The epilepsy genes network consisted of multiple interactions, whereas NDD genes showed minimal interaction. Epilepsy genes associated with different ion channels and neuronal cell body membranes were closely related among and within pathways. However, NDD genes showed no interactions among different pathways.

## 4. Discussion

This study examined the clinical spectrum and the distribution of genetic etiologies of pediatric epilepsy patients with NDDs. A total of 129 causative genes were identified in 168 NDD patients with epilepsy. Based on the main disease annotation, we classified the genes into two categories (epilepsy genes and NDD genes). The genes showed differences in GO pathways enrichment and heterogeneity. There were some statistical differences in the major seizure type and the epilepsy syndrome between patients in each group. This observation may be attributed to the high-frequency mutations in epilepsy genes among patients with specific diseases, such as West syndrome, Dravet syndrome, and Lennox–Gastaut syndrome. Other seizure phenotypes, including seizure onset age and DRE, showed no significant difference between the two groups. In addition, all neurologic features showed no significant difference between the two groups. Therefore, overlapping symptoms could make it difficult to differentiate the two groups based on only patients' symptoms. Complex and overlapping phenotype–genotype associations have led to the concept of DEE; our study demonstrated the validity of this concept and suggested future directions for genetic testing. Although detailed phenotyping is still essential when considering genetic testing in certain cases, clinical features may not provide sufficient information on tiered NGS data. Early NGS, possibly due to easy accessibility and reduced costs, may also accentuate the heterogeneous nature of patient phenotypes. Therefore, a phenotype-oriented genetic approach may not provide sufficient diagnostic clues when evaluating epilepsy and NDDs. There are few exceptions for typical early-onset epilepsy syndromes, such as Dravet syndrome or Ohtahara syndrome.

Patients with the same genetic etiology in the clinic can present complex and overlapping clinical courses that cannot be classified as biallelic disorders but are instead on a phenotypic continuum of NDDs. A patient with a *de novo* variant in *CACNA1A* (c.2413G>A, p. A712T) showed a typical DEE phenotype with very early-onset seizures (postnatal 1 month) and a poor response to anti-seizure drugs, whereas another patient with a different *de novo CACNA1A* variant (c.4031C>A, p. S1344Y) showed episodic ataxia and progressive cerebellar atrophy with juvenile-onset seizures (15 years old) and responded well to anti-seizure drugs. In the “NDD-genes” group, one patient with a *de novo* variant in *ABCC8* (c.257T>G, pV86G) presented with neonatal-onset diabetes mellitus, hypotonia, and frequent and prolonged seizures unrelated to hypoglycemia or hyperglycemia; nevertheless, although the patient's seizures were subsequently well controlled later. Another patient with compound heterozygous variants in *ABCC8* (c.2506C>T;c.2764C>T, p.R836X;p. Q922X) presented with congenital hyperinsulinemia accompanied by juvenile-onset seizures and a good response to drugs. Therefore, the different functional effects of variants in the same gene might influence phenotypic diversity. However, in this study, there was a limited number of patients with variants in the same gene, and the effects might be minimal. Furthermore, elucidating the genetic etiology according to the functional effects of variants is beyond the scope of our research.

In the “NDD-genes” group, there were 13 genes (ADADVL, HDAC8, ITPR1, NFIX, OGT, PTPN1, RAB3GAP1, SETD5, SMC3, SLC18A3, SPTBN2, THOC6, and WDR81) without definite reports of seizure-related phenotypes. Seven genes (*HDAC8, NFIX, OGT, RAB3GAP1, SETD5, SMC3*, and *THOC6*) could be predicted to cause seizures despite a lack of strong evidence. *HDAC8* is the causative gene for Cornelia de Lange syndrome (CdLS), a genetically heterogeneous disease entity with characteristic facial features, developmental delay, and other neurologic features. Although there have been no consistent reports of CdLS patients with HDAC8 variants and epileptic seizures, *SMC1* annotated to CdLS 2 (MIM#301044) is also designated DEE 85 (MIM#301044). In contrast, *ITPR1, PTPN1, SPTBN2*, and *WDR81* are known as cerebellar ataxia-related genes, and there is limited evidence of shared mechanisms with epilepsy. *ACADVL* and *HEPHL1* appear to have the weakest association with epilepsy and require further studies. The incidental occurrence of epilepsy in the “NDD-genes” group highlights the diversity of genotypes and phenotypes in epilepsy and NDDs and blurs the boundaries between the two disorders.

Interestingly, GO analysis revealed some differences between the two groups. Epilepsy genes associated with various ion channel complexes and neurotransmitter pathways showed dense interactions. Except for the AMPA glutamate receptor complex, which is associated with synaptic transmission, the biological networks of NDD genes were mostly associated with fundamental biological functions and structures, including DNA methylation, peroxisomal membrane function, selective autophagy, or head morphogenesis. Epilepsy genes showed compact and dense interactions with each other, whereas NDD genes showed a lack of interactions. The results are consistent with recent studies in which the molecular basis of epilepsy genes in NDD patients was analyzed (24). Various ion channel genes have been identified in early-onset epilepsy patients in the early stages of clinical genetic studies (25). Initial studies on the genetic etiology of neurologic diseases often focused on early-onset epilepsy as it shows an apparent phenotype. Genes associated with sodium or potassium channels were documented first as they are often involved in very early-onset seizures. Studies eventually progressed to channelopathy research in the field of genetic epilepsy followed. As the NGS technique has become widely adopted, research on broad or non-specific NDDs has identified different causative genes, and follow-up reports of seizure phenotypes have been published. Our GO analysis was based on the accumulated evidence. Channelopathy, the main disease entity identified in the GO analysis of epilepsy genes, is characterized by alterations in neuronal excitability. NDD genes showed limited interactions with each other; thus, these genes may be involved in several pathomechanisms. However, recent studies suggested that the underlying biological mechanisms of epilepsy and NDDs include the complex interactions of various biological dimensions, including genes, epigenomes, cells, brain functions, and clinical manifestations (26, 27). The findings of our study showing the similar clinical features of the two groups in our study supports the hypothesis that epilepsy and NDD are complex disorders that share neurodevelopmental processes. Further studies using advanced computational approaches, including integrative analysis of multiple biologic factors using omics data analysis, could shed light on the basic mechanisms underlying epilepsy and NDDs (28–30).

Our findings highlight overlapping neurologic features across different gene groups in an NDD cohort with epilepsy. We observed that various genes could be linked to different disease entities, including classic epilepsy syndromes, DEE, and neurodevelopmental disorders. These causative genes could be categorized based on their biological or molecular pathways, and the specific disease entity they are associated with. However, it is essential to note that patients carrying these genetic variants may exhibit heterogeneous and overlapping clinical courses in clinical practice. Considering the broad spectrum of phenotypes and genotypes in the NDD cohort, an exome- or genome-wide genetic approach would be preferable over a narrow-targeted approach based on phenotype except in cases with a highly suggestive etiology. The involvement of several causative genes involved in diverse molecular pathways and shared phenotypes demonstrated the complex and integrated mechanisms in the NDD cohort, which warrants further investigation.

## Data availability statement

The original contributions presented in the study are included in the article/Supplementary material, further inquiries can be directed to the corresponding author.

## Ethics statement

The studies involving human participants were reviewed and approved by Seoul National University Hospital Institutional Review Board. Written informed consent from the participants' legal guardian/next of kin was not required to participate in this study in accordance with the national legislation and the institutional requirements.

## Author contributions

YK and SK contributed to the study design, analyzed and interpreted the data, and drafted the manuscript for content. SL and HK contributed to the data analysis and interpreted the data. JY and MK contributed to the variant scoring. HJ and KK contributed to the gene ontology analysis. J-HC and KJK contributed to the study concepts. BL contributed to the study design and provided study supervision. All authors have read and agreed to the published version of the manuscript.
