# Supplementary material for: Epilepsy phenotype and gene ontology analysis of the 129 genes in a large neurodevelopmental disorders cohort
Source: Front Neurol. 2023 Aug 14;14:1218706. doi: 10.3389/fneur.2023.1218706 (PMC10461058; doi:10.3389/fneur.2023.1218706)
Supplement: Supplementary file 1 [file Table_1.docx]

Supplementary Material

Epilepsy phenotype and gene ontology analysis of the 129 genes in a large neurodevelopmental disorders cohort

Young Jun Ko^1†^, Soo Yeon Kim^2, 3†^, Seungbok Lee^3^, Jihoon G. Yoon^3^, Hyeji Jun^4^, Hunmin Kim^5^, Jong-Hee Chae^2, 3^, Ki Joong Kim^2^, Kwangsoo Kim^5^, Byung Chan Lim^2*^

*** Correspondence:** Hunmin Kim: hunminkim@hanmail.net

**Supplementary Table 1. Summary of variants from 67 patients in epilepsy-genes group.**

| **Case** | **Sex** | **Gene** | **Inheritance** | **Nucleotide change** | **Amino acid change** | **Variants** | **ACMG criteria** | **Pathogenicity** | **CADD** | **REVL** | **Splice** |
| --- | --- | --- | --- | --- | --- | --- | --- | --- | --- | --- | --- |
| Case 1 | F | ALDH5A1 | AR | c.1294A>C | p.Met432Leu | comhet | PS1 PM2 | LP | 26.1 | 0.877 |  |
|  |  |  | AR | c.1565A>G | p.Asp522Gly | comhet | PM2 PM3 PP3 PP4 | LP | 32 | 0.654 |  |
| Case 2 | M | ALDH7A1 | AR | c.1279G>C | p.Glu427Gln | comhet | PS3 PM2 PM5 PP3 PP5 | P | 28.9 | 0.944 |  |
|  |  |  | AR | c.1016A>G | p.His339Arg | comhet | PM1 PM2 PM3 | LP | 23.5 | 0.868 |  |
| Case 3 | F | ALDH7A1 | AR | c.1279G>C | p.Glu427Gln | homo | PS3 PM2 PM5 PP3 PP5 | P | 28,9 | 0.944 |  |
| Case 4 | F | ALG13 | XD | c.320A>G | p.Asn107Ser | de novo | PS2 PM1 PM2 PP4 PP5 | P | 21.8 | 0.344 |  |
| Case 5 | M | ARX | XR | c.196G>A | p.Gly66Ser | de novo | PS2 PM2 | LP | 28.3 | 0.271 |  |
| Case 6 | M | ARX | XR | c.989G>A | p.Arg330His | hemi | PM1 PM2 PP3 PP4 PP5 | LP | 26.7 | 0.944 |  |
| Case 7 | M | ARX | XR | c.1621G>T | p.Glu541Ter | hemi | PVS1 PM2 PP3 PP4 | P | 39 |  |  |
| Case 8 | F | CACNA1A | AD | c.2143G>A | p.Ala712Thr | de novo | PS2 PM1 PM2 PP3 PP5 | P | 29.3 | 0.952 |  |
| Case 9 | F | CACNA1A | AD | c.4031C>A | p.Ser1344Tyr | de novo | PS2 PM1 PM2 PM5 | P | 27.9 | 0.954 |  |
| Case 10 | F | CASK | XD | c.173-1G>C | - | de novo | PVS1 PS2 | P | 33 |  | 0.97 |
| Case 11 | F | CDKL5 | XD | c.446_447dup | p.Lys150fs | de novo | PS2 PM2 | LP |  |  |  |
| Case 12 | F | CDKL5 | XD | c.1684C>T | p.Arg550Ter | de novo | PVS1 PS2 | P | 33 |  |  |
| Case 13 | M | CLN6 | AR | c.707A>G | p.Arg62Cys | comhet | PM1 PM2 PP3 PP4 | LP | 32 | 0.926 |  |
|  |  |  | AR | c.806C>T | p.Thr269Ile | comhet | PM1 PM2 PP3 PP4 | LP | 24.1 | 0.736 |  |
| Case 14 | M | CLN6 | AR | c.784C>T | p.Leu262Phe | comhet | PM1 PM2 PP3 PP4 | LP | 25.9 | 0.675 |  |
|  |  |  | AR | c.806C>T | p.Thr269Ile | comhet | PM1 PM2 PP3 PP4 | LP | 24.1 | 0.736 |  |
| Case 15 | M | COL4A1 | AD | c.3281G>A | p.Gly1094Glu | NA | PM2 PM5 PP3 PP4 | LP | 25.7 | 0.968 |  |
| Case 16 | M | COL4A1 | AD | c.2850A>T | p.Lys950Asn | de novo | PS2 PM2 | LP | 22.8 | 0.585 |  |
| Case 17 | M | CYFIP2 | AD | c.259C>T | p.Arg87Cys | de novo | PS2 PM1 PM2 PP2 PP5 | P | 18.19 | 0.602 |  |
| Case 18 | F | CYFIP2 | AD | c.260G>T | p.Arg87Leu | NA | PM1 PM2 PP2 PP4 PP5 | LP | 19.66 | 0.688 |  |
| Case 19 | F | DNM1 | AD | c.431C>T | p.Pro144Leu | de novo | PS2 PM2 PP4 PP5 | LP | 29.4 | 0.891 |  |
| Case 20 | F | DNM1 | AD | c.709C>T | p.Arg237Trp | de novo | PS2 PM2 PP4 PP5 | LP | 24.7 | 0.928 |  |
| Case 21 | F | DYNC1H1 | AD | c.9721A>G | p.Lys3241Glu | de novo | PS2 PM1 PM2 PM5 | P | 23.6 | 0.544 |  |
| Case 22 | F | FGF12 | AD | c.341G>A | p.Arg114His | de novo | PS2 PM1 PM2 PP2 PP5 | P | 29.9 | 0.548 |  |
| Case 23 | M | FOXG1 | AD | c.412delG | p.Ala138fs | de novo | PVS1 PS2 | P |  |  |  |
| Case 24 | M | GABBR2 | AD | c.1699G>A | p.Ala567Thr | de novo | PS2 PM1 PM2 PP2 PP5 | P | 29.1 | 0.749 |  |
| Case 25 | F | GABRB1 | AD | c.1306C>T | p.Leu436Phe | de novo | PS2 PM1 PM2 PP3 PP4 | P | 25.7 | 0.483 |  |
| Case 26 | F | GNAO1 | AD | c.680C>T | p.Ala227Val | de novo | PS2 PM1 PM2 PP2 PP5 | P | 27.8 | 0.944 |  |
| Case 27 | F | GRIN1 | AD | c.1922T>A | p.Met641Lys | de novo | PS2 PM1 PM2 PM5 | P | 28.4 | 0.744 |  |
| Case 28 | F | GRIN2B | AD | c.2079A>T | p.Arg693Thr | de novo | PS2 PM1 PM2 | LP | 29 | 0.525 |  |
| Case 29 | F | GRIN2D | AD | c.2489A>G | p.Asn830Ser, | de novo | PS2 PM1 PM2 | LP | 22.4 | 0.172 |  |
|  |  |  | AD | c.2530A>C | p.Asn844His | de novo | PS2 PM1 PM2 | LP | 25.4 | 0.342 |  |
| Case 30 | M | IQSEC2 | XD | c.2139delC | p.Gly714fs | de novo | PVS1 PS2 | P |  |  |  |
| Case 31 | M | IQSEC2 | XD | c.918_922dupGAAGC | - | de novo | PVS1 PS2 | P |  |  |  |
| Case 32 | F | IQSEC2 | XD | c.4126_4127dupC | p.Gln1376fs | de novo | PVS1 PS2 | P |  |  |  |
| Case 33 | F | KCNB1 | AD | c.949C>T | p.Leu317Phe | NA | PM1 PM2 PP3 PP4 | LP | 25 | 0.923 |  |
| Case 34 | M | KCNC1 | AD | c.1262C>T | p.Ala421Val | NA | PS3 PM1 PM2 PP2 PP5 | P | 26 | 0.917 |  |
| Case 35 | M | KCNC1 | AD | c.959G>A | p.Arg320His | de novo | PS2 PM1 PM2 PP2 PP5 | P | 28.5 | 0.98 |  |
| Case 36 | F | KCNQ2 | AD | c.1054_1055delTC | p.Ser352fs | de novo | PVS1 PS2 | P |  |  |  |
| Case 37 | M | KCNQ2 | AD | c.1078T>G | p.Trp360Gly | de novo | PS2 PM1 PM2 PM5 | P | 32 | 0.953 |  |
| Case 38 | F | KCNT2 | AD | c.2534T>G | p.Met845Arg | de novo | PS2 PM1 PM2 PP2 PP4 | P | 26.7 | 0.906 |  |
| Case 39 | F | MECP2 | XD | c.910C>T | p.Leu313Phe | hemi | PM1 PM2 PP3 PP4 | LP | 25.9 | 0.494 |  |
| Case 40 | F | MECP2 | XD | c.916C>T | p.Arg306Ter | de novo | PVS1 PS2 | P | 39 |  |  |
| Case 41 | F | PACS2 | AD | c.625G>A | p.Glu209Lys | de novo | PS2 PM1 PM2 PP2 PP5 | P | 28.9 | 0.228 |  |
| Case 42 | F | PCDH19 | XD | c.2656C>T | p.Arg886Ter | de novo | PVS1 PS2 | P | 41 |  |  |
| Case 43 | M | PIGA | XR | c.191G>C | p.Gly64Ala | NA | PM1 PM2 PP2 PP3 PP4 | LP | 25.9 | 0.228 |  |
| Case 44 | F | PIGT | AR | c.250G>T | p.Glu84Ter | comhet | PVS1 PM2 PP3 PP5 | P | 39 |  |  |
|  |  |  | AR | c.1582G>A | p.Val528Met | comhet | PS3 PM1 PM2 PM3 | P | 31 | 0.588 |  |
| Case 45 | M | PPP3CA | AD | c.1268_1271dupTGAA | Lys424fs | de novo | PVS1 PS2 | P |  |  |  |
| Case 46 | F | PPP3CA | AD | c.844G>A | p.Glu282Lys | NA | PM1 PM2 PP4 PP5 | LP | 32 | 0.467 |  |
| Case 47 | M | SCN1A | AD | c.2671G>A | p.Gly891Arg | de novo | PS2 PM1 PM2 PP3 PP4 | P | 25.8 | 0.937 |  |
| Case 48 | M | SCN1B | AD | c.253C>T | p.Arg85Cys | NA | PM1 PM2 PP3 PP4 | LP | 26.8 | 0.828 |  |
| Case 49 | M | SCN2A | AD | c.4309-2A>G | - | de novo | PVS1 PS2 | P | 34 |  |  |
| Case 50 | F | SCN2A | AD | c.718G>C | p.Ala240Pro | de novo | PS2 PM1 PM2 PM5 | P | 26.5 | 0.909 |  |
| Case 51 | F | SCN2A | AD | c.2671A>G | p.Ile891Val | de novo | PS2 PM1 PM2 PP3 PP4 | P | 24.3 | 0.724 |  |
| Case 52 | F | SCN8A | AD | c.2549G>A | p.Arg850Gln | de novo | PS2 PM1 PM2 PP3 PP4 | P | 29.3 | 0.967 |  |
| Case 53 | M | SLC2A1 | AD | c.997C>T | p.Arg333Trp | NA | PS3 PM1 PM2 PP3 PP5 | P | 32 | 0.876 |  |
| Case 54 | M | SLC2A1 | AD | c.391delC | p.Val131fs | de novo | PVS1 PS2 | P |  |  |  |
| Case 55 | F | SLC6A1 | AD | c.1070C>T | p.Ala357Val | de novo | PS2 PM1 PM2 PP3 PP5 | P | 29.2 | 0.915 |  |
| Case 56 | F | SMC1A | XD | c.3103C>T | p.Arg1035Ter | de novo, | PVS1 PS2 | P | 34 |  |  |
| Case 57 | F | SMC1A | XD | c.3285+1G>C | - | de novo | PVS1 PS2 | P | 34 |  | 0.17 |
| Case 58 | F | SMC1A | XD | c.327_329delCAA | p.Lys88del | de novo | PS2 PM1 PM2 | LP |  |  |  |
| Case 59 | M | SPTAN1 | AD | c.4683T>G | p.Asp1561Glu | de novo | PS2 PM1 PM2 | LP | 22.4 |  |  |
| Case 60 | F | SPTAN1 | AD | c.6619_6621delGAG | p.Glu2207del | de novo | PS2 PS3 PM2 | P |  |  |  |
| Case 61 | M | STXBP1 | AD | c.1497C>G | p.Tyr499Ter | de novo | PVS1 PS2 | P | 35 |  |  |
| Case 62 | F | SYNGAP1 | AD | c.1219delC | p.Gln407fs | de novo | PVS1 PS2 | P |  |  |  |
| Case 63 | M | SYNGAP1 | AD | c.1513T>C | p.Tyr505His | de novo | PS2 PM1 PM2 | LP | 27.6 | 0.646 |  |
| Case 64 | M | SZT2 | AR | c.137C>T | p.Thr46Ile | comhet | PM1 PM2 PP3 PP4 | LP | 16.37 | 0.027 |  |
|  |  |  | AR | c.348C>G | p.Ile116Met | comhet | PM1 PM2 PM3 | LP | 15.97 | 0.097 |  |
| Case 65 | F | UGDH | AR | c.1364T>C | p.Ile455Thr | comhet | PM1 PM2 PP2 PP4 | LP | 23.6 | 0.732 |  |
|  |  |  | AR | c.883G>T | p.Glu295Ter | comhet | PVS1 PM2 PP3 PP4 | P | 45 |  |  |
| Case 66 | M | YWHAG | AD | c.169C>T | p.Arg57Cys | de novo | PS2 PM1 PM2 PP2 PP4 | P | 29.9 | 0.891 |  |
| Case 67 | M | YWHAG | AD | c.394C>T | p.Arg132Cys | de novo | PS2 PM1 PM2 PP2 PP5 | P | 30 | 0.791 |  |

M, male; F, female; AR, autosomal recessive; AD, autosomal dominant, XR, X-linked recessive; XD, X-linked dominant; homo, homozygous variants; comhet; compound heterozygous variants; NA, not applicable; LP, likely pathogenic; P, pathogenic

**Supplementary Table 2. Summary of variants from 101 patients in NDD-genes group.**

| **Case** | **Sex** | **Gene** | **Inheritance** | **Nucleotide change** | **Amino acid change** | **Variants** | **ACMG criteria** | **Pathogenicity** | **CADD** | **REVL** | **SpliceAI** |
| --- | --- | --- | --- | --- | --- | --- | --- | --- | --- | --- | --- |
| Case 68 | M | ABAT | AR | c.1417C>T homo | p.Arg473Cys | homo | PM1 PM2 PP3 PP4 | LP | 32 | 0.93 |  |
| Case 69 | M | ABCC8 | AR | c.2506C>T | p.Arg836Ter | comhet | PVS1 PM2 PP3 PP4 PP5 | P | 41 |  |  |
|  |  |  | AR | c.2764C>T | p.Gln922Ter | comhet | PVS1 PM2 PP3 PP4 PP5 | P | 43 |  |  |
| Case 70 | M | ABCC8 | AD | c.257T>G | p.Val86Gly | de novo | PS2 PM1 PM2 PP3 PP4 PP5 | P | 27.1 | 0.97 |  |
| Case 71 | M | ACO2 | AR | c.1179G>A | p.Met393Ile | comhet | PM1 PM2 PP2 PP4 | LP | 23.2 | 0.359 |  |
|  |  |  | AR | c.1343G>C | p.Cys448Ser | comhet | PM1 PM2 PP2 PP3 PP4 | LP | 28.8 | 0.948 |  |
| Case 72 | F | ACOX1 | AD | c.710A>G | p.Asn237Ser | de novo | PS1 PS2 | P | 26 | 0.864 |  |
| Case 73 | M | ANKRD11 | AD | c.3224_3227del | p.Glu1075fs | de novo | PVS1 PS2 PP4 PP5 | P |  |  |  |
| Case 74 | F | ARID1B | AD | c.2872C>T | p.Gln958Ter | NA | PVS1 PM2 PP3 PP4 | LP | 42 |  |  |
| Case 75 | M | ARID1B | AD | c.5086G>T | p.Gly1696Ter | NA | PVS1 PM2 PP3 PP4 | LP | 37 |  |  |
| Case 76 | F | ARSA | AR | c.416_465+13del | p.P137_T157del | comhet | PM2 PM3 PM4 | LP | 28 | 0.959 |  |
|  |  |  | AR | c.302G>T | p.Gly101Asp | comhet | PM2 PM5 PP3 PP4 PP5 | LP |  |  |  |
| Case 77 | M | ASXL1 | AD | c.2775_2785delinsT | p.Val926fs | de novo | PS2 PM2 PP3 PP4 | LP |  |  |  |
| Case 78 | F | ATP6V0A2 | AR | c.294+1G>T homo | -/- | homo | PVS1 PM2 PP4 PP5 | P | 34 |  | 0.34 |
| Case 79 | M | ATRX | XD | c.5461A>G | p,Thr1821Ala | hemi | PM1 PM2 PP3 PP4 | LP | 24.4 | 0.616 |  |
| Case 80 | M | BRAF | AD | c.1593G>C | p.Trp531Cys | de novo | PS2 PM1 PM2 PP3 PP4 PP5 | P | 32 | 0.857 |  |
| Case 81 | F | BRAT1 | AR | c.1276C>T | p.Glu426Ter | comhet | PVS1 PM2 PP3 PP5 | P | 24 | 0.702 |  |
|  |  |  | AR | c.1684C>G | p.Arg562Gly | comhet | PM2 PM3 PM5 | LP | 43 |  |  |
| Case 82 | F | BRAT1 | AR | c.1276C>T | p.Gln426Ter | comhet | PVS1 PM2 PP3 PP4 | P | 43 |  |  |
|  |  |  | AR | c.1313_1314del | p.Gln438fs | comhet | PVS1 PM2 PP4 PP5 | P |  |  |  |
| Case 83 | M | CAMK2A | AD | c.635C>G | p.Pro212Arg | de novo | PS2 PM1 PM2 PM5 | P | 28.3 | 0.699 |  |
| Case 84 | M | COX15 | AR | c.664C>T | p.Arg222Cys | homo | PM1 PM2 PP3 PP4 | LP | 29.7 | 0.886 |  |
| Case 85 | M | CSNK2B | AD | c.101T>A | p.Phe34Tyr | de novo | PS2 PM1 PM2 PP3 PP4 | P | 27.9 | 0.815 |  |
| Case 86 | F | DDX3X | XD | c.1423C>T | p.Arg475Cys | hemi | PM1 PM2 PP3 PP4 PP5 | LP | 25.4 | 0.714 |  |
| Case 87 | M | DEGS1 | AR | c.265C>G | p.His89Asp | comhet | PM1 PM2 PP3 PP4 | LP | 27.1 | 0.89 |  |
|  |  |  | AR | c.729_732delCTCA | p.Ser244fs | comhet | PVS1 PM2 PM3 | LP |  |  |  |
| Case 88 | F | DHDDS | AD | c.110G>A | p.Arg37His | de novo | PS2 PM1 PM2 PP2 PP3 PP5 | P | 32 | 0.857 |  |
| Case 89 | M | DHDDS | AD | c.614G>A | p.Arg205Gln | de novo | PS2 PM1 PM2 PP2 PP3 PP5 | P | 32 | 0.825 |  |
| Case 90 | M | DHDDS | AD | c.632G>A | p.Arg211Gln | de novo | PS2 PM1 PM2 PP2 PP3 PP5 | P | 32 | 0.889 |  |
| Case 91 | M | DLG4 | AD | c.171C>A | p.Tyr57Ter | de novo | PVS1 PS2 | P | 36 |  |  |
| Case 92 | M | DNM1L | AR | c.1223C>A | p.Ala408Asp | homo | PM1 PM2 PP3 PP4 | LP | 29.7 | 0.874 |  |
| Case 93 | M | DNMT3A | AD | c.2644C>T | p.Arg882Cys | NA | PM1 PM5 PP3 PP5 | LP | 32 | 0.89 |  |
| Case 94 | F | DYRK1A | AD | c.1529delC | p.Ala510fs | de novo | PVS1 PS2 | P |  |  |  |
| Case 95 | F | EIF2AK2 | AD | c.325G>T | p.Ala109Ser | NA | PM1 PM2 PP2 PP4 | LP | 0.591 | 0.264 |  |
| Case 96 | F | EIF2B2 | AR | c.254T>A | p.Val85Glu | comhet | PM2 PM3 PP3 PP4 PP5 | LP | 30 |  |  |
|  |  |  | AR | c.677T>A | p.Met226Lys | comhet | PM1 PM2 PP3 PP4 PP5 | LP | 31 |  |  |
| Case 97 | M | EIF2S3 | XR | c.15A>C | p.Glu5Asp | hemi | PM2 PM6 PP3 PP4 | LP | 14.08 | 0.136 |  |
| Case 98 | F | GJA1 | AD | c.113G>A | p.Gly38Glu | de novo | PS2 PM1 PM2 PM5 | P | 24.5 | 0.932 |  |
| Case 99 | F | GLB1 | AR | c.203G>A | p.Arg68Gln | comhet | PM1 PM2 PP3 PP4 PP5 | LP | 32 | 0.975 |  |
|  |  |  | AR | c.1343A>T | p.Asp448Val | comhet | PM1 PM2 PP3 PP4 PP5 | LP | 25.5 | 0.832 |  |
| Case 100 | F | GLB1 | AR | c.517_519delCTC | p.Leu173del | comhet | PM1 PM2 PM3 | LP |  |  |  |
|  |  |  | AR | c.1343A>T | p.Asp448Val | comhet | PM1 PM2 PP3 PP4 PP5 | LP | 25.5 | 0.832 |  |
| Case 101 | F | GLB1 | AR | c.734-10C>G | - | comhet | PM2 PM3 PP3 PP4 | LP |  |  | 0.99 |
|  |  |  | AR | c.932G>A | p.Gly311Glu | comhet | PM1 PM2 PP3 PP4 PP5 | LP | 26 | 0.865 |  |
| Case 102 | M | GLB1 | AR | c.424A>T | p.Lys142Ter | comhet | PVS1 PM2 PP3 PP4 | P | 25.5 | 0.832 |  |
|  |  |  | AR | c.1343A>T | p.Asp448Val | comhet | PM1 PM2 PM3 | LP | 40 |  |  |
| Case 103 | F | GLB1 | AR | c.950A>T | Asp317Val | homo | PM1 PM2 PM5 | LP | 25.5 | 0.832 |  |
| Case 104 | M | GRIA2 | AD | c.1820A>T | p.Gln607Leu | de novo | PS2 PM1 PM2 PM5 | P | 23.8 | 0.374 |  |
| Case 105 | M | GRIA3 | XR | c.1858G>A | p.Gly620Arg | de novo | PS2 PM1 PM2 PP3 PP4 | P | 32 | 0.974 |  |
| Case 106 | M | HEXA | AR | c.1168C>T | p.Gln390Ter | comhet | PVS1 PM2 PP3 PP4 PP5 | P | 39 |  |  |
|  |  |  | AR | c.571-1G>T | - | comhet | PVS1 PM2 PM3 PP4 PP5 | P | 34 |  |  |
| Case 107 | F | HEXA | AR | c.1A>C | p.Met1Leu | comhet | PM1 PM2 PM3 PM5 | LP | 22.9 | 0.567 |  |
|  |  |  | AR | c.1168C>T | p.Gln390Ter | comhet | PVS1 PM2 PP3 PP4 PP5 | P | 39 |  |  |
| Case 108 | M | HK1 | AD | c.1382C>T | p.Tyr461Met | de novo | PS2 PM1 PM2 PP2 PP4 PP5 | P | 31 | 0.358 |  |
| Case 109 | M | HSD17B4 | AR | c.350A>T | p.Asp117Val | comhet | PM1 PM2 PM3 | LP | 33 | 0.883 |  |
|  |  |  | AR | c.745T>G | p.Trp249Gly | comhet | PM1 PM2 PP3 PP4 PP5 | P | 25.3 | 0.887 |  |
| Case 110 | M | HUWE1 | AD | c.8077C>T | p.Arg2693Cys | de novo | PS2 PM1 PM2 PP4 | LP | 25.3 | 0.224 |  |
| Case 111 | M | IARS2 | AR | c.314_318delTAAA | p.Val105Aspfs*7 | comhet | PM2 PM3 PM4 | LP |  |  |  |
|  |  |  | AR | c.2450G>A | p.Arg817His | comhet | PM1 PM2 PP4 PP5 | LP | 26.2 | 0.569 |  |
| Case 112 | M | IARS2 | AR | c.971_972delCT | p.Ser324Ter | comhet | PVS1 PM2 PM3 | P |  |  |  |
|  |  |  | AR | c.2450G>A, | p.Arg817His | comhet | PM1 PM2 PP4 PP5 | LP | 26.2 | 0.569 |  |
| Case 113 | M | KDM5C | XR | c.1964delC | p.Pro655Glnfs*4 | hemi | PVS1 PM2 PM4 | P |  |  |  |
| Case 114 | M | KIAA1109 | AR | c.503C>T | p.Thr168Ile | comhet | PM1 PM3 PP2 PP4 | LP | 12.98 | 0.187 |  |
|  |  |  | AR | c.7549A>T | p.Thr2517Ser | comhet | PM1 PM2 PP2 PP4 | LP | 23.8 | 0.329 |  |
| Case 115 | M | KIF4A | XR | c.1325C>T | p.Ala442Val | hemi | PM2 PM6 PP3 PP4 | LP | 22.6 | 0.026 |  |
| Case 116 | M | KMT2A | AD | c.3503G>A | p.Gly1168Asp | de novo | PS2 PM1 PM2 PP3 PP5 | P | 28.3 | 0.829 |  |
| Case 117 | F | KMT2C | AD | c.9235C>T | p.Arg3079Ter | de novo | PVS1 PS2 | P | 44 |  |  |
| Case 118 | F | KMT2D | AD | c.7325delC | p.Pro2442Leufs*43 | - | PVS1 PM2 PP3 PP4 | P |  |  |  |
| Case 119 | M | L1CAM | XR | c.92T>C | p.Val31Ala | hemi | PM1 PM2 PP3 PP5 | LP | 20.8 |  |  |
| Case 120 | M | LAMA2 | AR | c.595T>C | p.Cys199Arg | comhet | PM2 PM3 PP2 PP4 | LP | 28 | 0.944 |  |
|  |  |  | AR | c.7605delT | p.Pro2535fs | comhet | PVS1 PM2 | LP |  |  |  |
| Case 121 | F | LONP1 | AR | c.426C>G | p.Ile142Met | comhet | PM1 PM2 PP3 PP4 | LP | 16.45 | 0.103 |  |
|  |  |  | AR | c.1081A>G | p.Lys361Glu | comhet | PM2 PM3 PP3 PP4 | LP | 23.2 | 0.359 |  |
| Case 122 | M | MAPK8IP3 | AD | c.1732C>T | p.Arg578Cys | de novo | PS2 PM1 PM2 PP3 PP5 | P | 29.2 | 0.675 |  |
| Case 123 | M | NALCN | AD | c.1733A>G | p.Tyr578Cys | de novo | PS2 PM2 PM5 PP3 PP5 | P | 29.8 | 0.976 |  |
| Case 124 | F | NARS2 | AR | c.731C>G | p.Ala244Gly | comhet | PM1 PM2 PP3 PP4 | LP | 27.7 | 0.869 |  |
|  |  |  | AR | c.1351C>T | p.Arg451Cys | comhet | PM1 PM2 PM3 | LP | 32 | 0.937 |  |
| Case 125 | M | NARS2 | AR | c.301A>G | p.Ser101Gl | comhet | PM2 PM3 PP2 PP4 | LP | 25.8 | 0.416 |  |
|  |  |  | AR | c.1026+2A>C | - | comhet | PVS1 PM2 PM3 | P | 33 |  | 0.77 |
| Case 126 | F | NDUFAF6 | AR | c.820A>G | p.Arg274Gly | comhet | PM1 PM2 PM3 | LP | 24.5 | 0.772 |  |
|  |  |  | AR | c.874-2A>C | - | comhet | PVS1 PM2 | LP | 34 |  |  |
| Case 127 | F | NDUFV1 | AR | c.733G>A | p.Val245Met | comhet | PM1 PM2 PP3 PP5 | LP | 27.4 | 0.856 |  |
|  |  |  | AR | c.1235T>C | p.Leu412Pro | comhet | PM1 PM2 PM3 | LP | 28.7 | 0.963 |  |
| Case 128 | F | NSD1 | AD | Exons 1~24 deletion* |  | NA | PVS1 PM2 PM4 | P |  |  |  |
| Case 129 | M | NSD1 | AD | c.4786T>C | p.Cys1596Arg | NA | PM1 PM2 PM5 | LP | 28.6 | 0.986 |  |
| Case 130 | M | NSD1 | AD | c.7259_7270delinsGT | p.Pro2420Argfs*11 | NA | PVS1 PM2 PM4 | P |  |  |  |
| Case 131 | M | OPHN1 | XR | c.67413783 C>A | p.Glu384Ter | hemi | PVS1 PM2 | LP | 45 |  |  |
| Case 132 | M | OTUD6B | AR | c.735C>G | p.Tyr245Ter | comhet | PVS1 PM2 | P | 36 |  |  |
|  |  |  | AR | c.626T>G | p.Leu209Trp | comhet | PM2 PM3 PP2 PP4 | LP | 23.6 | 0.519 |  |
| Case 133 | M | PAFAH1B1 | AD | c.934_940delCTGTCTG | p.Leu312Aspfs*1 | NA | PVS1 PM2 PP4 | P |  |  |  |
| Case 134 | F | PCYT2 | AR | c.730C>T | p.His244Tyr | homo | PM1 PM3 PP3 PP4 PP5 | LP | 28.1 | 0.98 |  |
| Case 135 | F | PDHA1 | XD | c.1142_1145dupATCA | p.Trp421fs | de novo | PVS1 PS2 | P |  |  |  |
| Case 136 | F | PEX5 | AR | c.1354C>T | p.Arg452Trp | comhet | PM1 PM2 PM3 | LP | 24.1 | 0.478 |  |
|  |  |  | AR | c.1615C>T | p.Arg539Cys | comhet | PM1 PM2 PP2 PP3 PP4 | LP | 32 | 0.7 |  |
| Case 137 | M | PIK3R2 | AD | c.1117G>A | p.Gly373Arg | de novo | PS1 PS2 | P | 25.3 | 0.642 |  |
| Case 138 | F | PLA2G6 | AR | c.668C>T | p.Pro223Leu | comhet | PM2 PM3 PP2 PP4 | LP | 29 | 0.888 |  |
|  |  |  | AR | c.1799G>A | p.Arg600Gln | comhet | PM1 PM2 PP3 PP5 | LP | 27.9 | 0.806 |  |
| Case 139 | M | PPP2R5D | AD | c.592G>A | p.Glu198Lys | de novo | PS1 PS2 | P | 40 | 0.571 |  |
| Case 140 | M | PRUNE1 | AR | c.1036delG homo | p.Val346fs | homo, | PM2 PM4 PP3 PP4 | LP |  |  |  |
| Case 141 | M | PTPN23 | AR | c.345delC | p.Cys116fs | comhet | PVS1 PM2 PP4 | P |  |  |  |
|  |  |  |  | c.4052A>G | p.His1351Arg | comhet | PM1 PM2 PM3 | LP | 23.5 | 0.237 |  |
| Case 142 | M | SETD2 | AD | c.3073G>A | p.Gly1025Ser | de novo | PS2 PM1 PM2 PP3 PP4 | P | 23.8 | 0.225 |  |
| Case 143 | M | SLC19A3 | AR | c.265A>C | p.Ser89Arg | comhet | PM1 PM2 PM3 | LP | 16.7 | 0.407 |  |
|  |  |  | AR | c.449C>T | p.Ala150Val | comhet | PM1 PM2 PP3 PP5 | LP | 24.9 | 0.658 |  |
| Case 144 | F | SNAP25 | AD | c.25A>G | p.Asn9Asp | de novo | PS2 PM1 PM2 PP2 PP4 | P | 23.9 | 0.288 |  |
| Case 145 | F | ST3GAL5 | AR | c.584G>C | p.Cys195Ser | comhet | PM1 PM2 PP3 PP5 | LP | 24.8 | 0.67 |  |
|  |  |  | AR | c.601G>A | p.Gly201Arg | comhet | PM1 PM2 PP4 PP5 | LP | 26.3 | 0.913 |  |
| Case 146 | F | TRRAP | AD | c.9977T>C | p.Val3326Ala | de novo | PS2 PM1 PM2 | LP | 23.1 | 0.287 |  |
| Case 147 | M | TUBA1A | AD | c.497A>G | p.Lys166Arg | de novo | PS2 PM1 PM2 PP2 PP3 | P | 25.4 | 0665 |  |
| Case 148 | F | TUBB4A | AD | c.158G>A | p.Arg53Gln | de novo | PS2 PM1 PM2 PP4 PP5 | P | 24.4 | 0.795 |  |
| Case 149 | M | TUBB4A | AD | c.293G>A | P.Gly98Asp | de novo | PS2 PM1 PM2 PP3 PP4 | P | 26.4 | 0.907 |  |
| Case 150 | F | TUBGCP6 | AR | c.4831C>T | p.Pro1611Ser | comhet | PM1 PM2 PP3 PP4 | LP | 25.5 | 0.559 |  |
|  |  |  | AR | c.5050A>G | p.Ile1684Val | comhet | PM2 PM3 PP3 PP4 | LP | 22.6 | 0.104 |  |
| Case 151 | M | UBE3A | AD | c.1844dupA | p.Leu619fs | de novo | PVS1 PS2 | P |  |  |  |
| Case 152 | F | UBE3A | AD | c.2549C>T | p.Pro850Leu | de novo | PS2 PM1 PM2 PP3 PP4 | P | 26.7 | 0.678 |  |
| Case 153 | F | VPS13B | AR | c.7876G>A | p.Glu2626Lys | comhet | PM1 PM2 PP2 PP4 | LP | 49 | 0.298 |  |
|  |  |  | AR | c.10312G>T | p.Ala3438Ser | comhet | PM1 PM2 PP2 PP4 | LP | 4.93 | 0.083 |  |
| Case 154 | M | VPS13D | AR | c.6170A>G | p.Lys2057Arg | comhet | PM1 PM2 PP3 PP4 | LP | 18.38 | 0.029 |  |
|  |  |  | AR | c.11097,G>A | p.Met3699Ile | comhet | PM1 PM2 PM3 | LP | 23.5 | 0.141 |  |
| Case 155 | F | WDR26 | AD | c.1839dupA | p.Arg614fs | de novo | PVS1 PS2 | P |  |  |  |
| Case 156 | F | ACADVL | AR | c.619T>C | p.Ser207Pro | comhet | PM1 PM2 PM3 | LP | 24.8 | 0.792 |  |
|  |  |  | AR | c.G1349A | p.Arg450His | comhet | PM1 PM2 PP3 PP5 | LP | 28.5 | 0.92 |  |
| Case 157 | F | HDAC8 | XD | c.145G>A | p.Ala49Thr | de novo | PS2 PM2 | LP | 23.3 | 0.185 |  |
| Case 158 | M | HEPHL1 | AR | c.3049_3052delGATA | p.Asp1017fs | comhet | PVS1 PP3 PP4 | P |  |  |  |
|  |  |  | AR | c.1678G>T | p.Val560Phe | comhet | PM1 PM2 PP3 PP4 | LP | 26.9 | 0.936 |  |
| Case 159 | M | ITPR1 | AD | c.3428G>T | p.Gly1143Val | probable | PM1 PM2 PM6 | LP | 17.78 | 0.426 |  |
| Case 160 | F | NFIX | AD | c.610C>T | p.Gln204Ter | de novo | PVS1 PS2 | P | 22.8 | 0.149 |  |
| Case 161 | F | OGT | XR | c.2444A>G | p.Asn815Ser | hemi | PM1 PM2 PP2 PP4 | LP | 14.72 | 0.199 |  |
| Case 162 | M | PNPT1 | AR | c.574C>T | p.Arg192Ter | comhet | PVS1 PM2 PP3 PP4 | P | 27.3 | 0.526 |  |
|  |  |  | AR | c.1520C>G | p.Ala507Gly | comhet | PM2 PM3 PP3 PP4 | LP | 38 |  |  |
| Case 163 | M | RAB3GAP1 | AR | c.691C>T | p.Arg231Ter | comhet | PVS1 PM2 PP3 PP5 | P | 38 |  |  |
|  |  |  | AR | c.1562dupA | p.Asn521Lysfs*5 | comhet | PVS1 PM2 PM3 | P |  |  |  |
| Case 164 | F | SETD5 | AD | c.2545delC | p.Arg849fs | de novo | PVS1 PS2 | P |  |  |  |
| Case 165 | F | SMC3 | AD | c.3476-2A>G | - | NA | PVS1 PM2 | P | 35 |  | 1 |
| Case 166 | M | SPTBN2 | AD | c.944C>T | p.Ser315Leu | Inherited from affected parent | PM1 PM2 PP1 PP3 | LP | 25.5 | 0.687 |  |
| Case 167 | F | WDFY3 | AD | c.655G>T | p.Ala219Ser | de novo | PS2 PM2 PP2 PP4 | P | 25.3 | 0.39 |  |
| Case 168 | F | WDR81 | AR | c.5083C>G | p.Gln1695Glu | comhet | PM1 PM2 PP2 PP4 | LP | 15.22 | 0.25 |  |
|  |  |  | AR | c.5294G>A | p.Arg1765His | comhet | PM1 PM2 PP2 PP4 | LP | 20.2 | 0.381 |  |

M, male; F, female; AR, autosomal recessive; AD, autosomal dominant, XR, X-linked recessive; XD, X-linked dominant; homo, homozygous variants; comhet; compound heterozygous variants; NA, not applicable; LP, likely pathogenic; P, pathogenic

* The identification of the deletion was initially achieved through exome sequencing data, and subsequent validation was carried out using multiplex ligation dependent probe amplification assay.

**Supplementary Table 3. Phenotype and genotype in patients with same causative genes**

| **Case** | **Sex** | **Gene** | **Seizure onset age, years** | **Initial  seizure type** | **DRE** | **Epilepsy syndromes** | **Neurological symptoms** |
| --- | --- | --- | --- | --- | --- | --- | --- |
| Case 69 | M | ABCC8 | 13 | FT | None | - | Severe ID, brain abnormality (diffuse WM change), microcephaly |
| Case 70 | M | ABCC8 | 0.1 | FT | None | - | none |
| Case 2 | M | ALDH7A1 | 0 | F | None | - | Hypotonia, Severe ID, regression |
| Case 3 | F | ALDH7A1 | 0.7 | S | None | WS | Severe ID, microcephaly |
| Case 74 | F | ARID1B | 1 | U | None | - | Hypotonia, ataxia, Severe ID, facial dysmorphism, brain abnormality (CCA) |
| Case 75 | M | ARID1B | 6 | U | None | - | Hypotonia, ataxia, Severe ID, regression, facial dysmorphism |
| Case 5 | M | ARX | 0 | U | Yes | WS | Severe ID, microcephaly, brain abnormality (LIS) |
| Case 6 | M | ARX | 0.2 | S | None | WS | Hypotonia, spasticity, Severe ID |
| Case 7 | M | ARX | 0.3 | S | Yes | WS | Hypotonia, dyskinesia, Severe ID, brain abnormality (CCA) |
| Case 81 | F | BRAT1 | 0.3 | F | Yes | - | Severe ID, regression, microcephaly |
| Case 82 | F | BRAT1 | 0 | GTC | Yes | - | Spasticity, Severe ID, microcephaly, brain abnormality (diffuse WM change) |
| Case 8 | F | CACNA1A | 0 | F | Yes | LGS | Severe ID |
| Case 9 | F | CACNA1A | 15 | F | None | - | Hypotonia, Mild ID, brain abnormality (cerebellar atrophy) |
| Case 11 | F | CDKL5 | U | U | Yes | - | Hypotonia, Severe ID, ASD, regression |
| Case 12 | F | CDKL5 | 0.3 | S | Yes | - | Ataxia, Severe ID |
| Case 13 | M | CLN6 | 10 | F | Yes | - | Spasticity, Mild ID, regression, brain abnormality (diffuse WM change, atrophy) |
| Case 14 | M | CLN6 | 7 | M | Yes | - | Ataxia, regression, brain abnormality (progressive cerebral/cerebellar atrophy) |
| Case 15 | M | COL4A1 | 1.1 | S | Yes | - | Severe ID |
| Case 16 | M | COL4A1 | 0.3 | S | Yes | WS | Hypotonia, Severe ID, microcephaly, , brain abnormality (diffuse WM change) |
| Case 17 | M | CYFIP2 | 0.5 | H | None | - | Hypotonia, Severe ID, microcephaly |
| Case 18 | F | CYFIP2 | 1 | U | Yes | - | Dyskinesia, Severe ID, ASD, regression, microcephaly, brain abnormality (LIS) |
| Case 88 | F | DHDDS | 0.8 | F | None | - | Ataxia, Mild ID |
| Case 89 | M | DHDDS | 0.7 | F | None | - | Moderate ID |
| Case 90 | M | DHDDS | 1 | A | None | - | Hypotonia, ataxia, Moderate ID |
| Case 19 | F | DNM1 | 0.2 | F | Yes | WS | Severe ID, microcephaly |
| Case 20 | F | DNM1 | 0.6 | S | None | WS | Hypotonia, Severe ID |
| Case 99 | F | GLB1 | 1 | F | None | - | Hypotonia, Severe ID, regression, brain abnormality (diffuse hypomyelination) |
| Case 100 | F | GLB1 | 2 | M | Yes | - | Hypotonia, Severe ID, regression, macrocephaly, brain abnormality (diffuse hypomyelination) |
| Case 101 | F | GLB1 | 3 | T | Yes | - | Hypotonia, Severe ID, regression, brain abnormality (diffuse hypomyelination) |
| Case 102 | M | GLB1 | 0.9 | F | Yes | - | Hypotonia, Severe ID, regression, brain abnormality (diffuse hypomyelination) |
| Case 103 | F | GLB1 | 1.3 | F | None | - | Hypotonia, Severe ID, brain abnormality (diffuse hypomyelination) |
| Case 106 | M | HEXA | 1.2 | F | Yes | - | Hypotonia, Severe ID, regression, macrocephaly, brain abnormality (diffuse WM change) |
| Case 107 | F | HEXA | 2 | T | Yes | - | Spasticity, Severe ID, regression, brain abnormality (diffuse WM change) |
| Case 111 | M | IARS2 | 5 | S | None | - | Hypotonia, spasticity, dyskinesia, Moderate ID, regression, microcephaly, brain abnormality (diffuse WM change) |
| Case 112 | M | IARS2 | 2.3 | S | Yes | - | Hypotonia, Mild ID, microcephaly, brain abnormality (diffuse WM change) |
| Case 30 | M | IQSEC2 | 1.7 | T | Yes | LGS | Severe ID, regression, macrocephaly |
| Case 31 | M | IQSEC2 | 5 | M | None | - | Hypotonia, ataxia, Moderate ID, ASD, microcephaly |
| Case 32 | F | IQSEC2 | 1.5 | GTC | None | - | Moderate ID |
| Case 34 | M | KCNC1 | 12 | GTC | Yes | - | Ataxia, regression, brain abnormality (cerebellar atrophy) |
| Case 35 | M | KCNC1 | 0.6 | M | None | - | Mild ID |
| Case 36 | F | KCNQ2 | 0.3 | U | None | - | Mild ID, ASD, regression |
| Case 37 | M | KCNQ2 | 0 | F | None | - | Hypotonia, Mild ID |
| Case 39 | F | MECP2 | 12 | U | None | - | Ataxia, Mild ID, ASD, regression, |
| Case 40 | F | MECP2 | 4 | GTC | None | - | Ataxia, Moderate ID, ASD, regression |
| Case 124 | F | NARS2 | 0.3 | F | Yes | - | Spasticity, dyskinesia, Mild ID, regression, brain abnormality (bilateral basal ganglia change) |
| Case 125 | M | NARS2 | 0.3 | F | Yes | - | Hypotonia, spasticity, Mild ID, regression, brain abnormality (diffuse WM change) |
| Case 128 | F | NSD1 | 6 | Ab | Yes | CAE | Mild ID, macrocephaly |
| Case 129 | M | NSD1 | 13 | T | None | - | Mild ID, macrocephaly |
| Case 130 | M | NSD1 | 2 | T | None | - | Hypotonia, ataxia, Mild ID, macrocephaly |
| Case 45 | M | PPP3CA | 0.2 | M | Yes | WS | Hypotonia, spasticity, Severe ID |
| Case 46 | F | PPP3CA | 2.5 | GTC | Yes | LGS | Moderate ID, facial dysmorpism |
| Case 50 | M | SCN2A | 2.5 | GTC | Yes | - | Moderate ID |
| Case 51 | F | SCN2A | 0.5 | M | None | - | Moderate ID |
| Case 52 | F | SCN2A | 15 | GTC | None | - | Mild ID |
| Case 54 | M | SLC2A1 | 2 | GTC | None | - | Ataxia, microcephaly |
| Case 55 | M | SLC2A1 | 1 | T | None | - | Dyskinesia, Mild ID |
| Case 56 | F | SMC1A | 1 | F | Yes | - | Hypotonia, Mild ID, facial dysmorphism, microcephaly |
| Case 57 | F | SMC1A | 0.2 | GTC | Yes | WS | Hypotonia, Mild ID, facial dysmorphism, microcephaly, brain abnormality (LIS) |
| Case 58 | F | SMC1A | 0.5 | S | Yes | WS, LGS | Moderate ID, regression |
| Case 62 | M | SPTAN1 | 0 | T | Yes | - | Spasticity, Severe ID, facial dysmorphism, microcephaly, brain abnormality (Diffuse hypomyelination, cerebellar atrophy) |
| Case 63 | F | SPTAN1 | 1.4 | GTC | None | - | Severe ID |
| Case 62 | F | SYNGAP1 | 1.5 | Ab | None | - | Severe ID |
| Case 63 | M | SYNGAP1 | 20 | M | None | - | Dyskinesia, Moderate ID |
| Case 148 | F | TUBB4A | 3 | M | Yes | - | Hypotonia, spasticity, Moderate ID, regression, brain abnormality (Diffuse hypmyelination) |
| Case 149 | M | TUBB4A | 1 | U | None | - | Hypotonia, spasticity, ataxia, Moderate ID, regression, brain abnormality (Diffuse hypmyelination) |
| Case 151 | M | UBE3A | 3 | A | None | - | Mild ID, facial dysmorphism |
| Case 152 | F | UBE3A | 1.9 | A | None | - | Ataxia, Mild ID |
| Case 66 | M | YWHAG | 0.3 | F | Yes | - | Moderate ID |
| Case 67 | M | YWHAG | 2 | T | None | - | Moderate ID |

A, atonic seizure; Ab, absence seizure; CAE, childhood absence epilepsy; CCA, corpus callosal agenesis; GTC, generalized tonic-clonic seizure; H, hemiclonic; ID, intellectual disability; LGS, Lennox-Gastaut syndrome; LIS, lissencephaly; M, myoclonic seizure; S, spasm; T, tonic seizure; U, unknown; WS, West syndrome
